# The Frequent Stressor and Mental Health Monitoring-Paradigm: A Proposal for the Operationalization and Measurement of Resilience and the Identification of Resilience Processes in Longitudinal Observational Studies

**DOI:** 10.3389/fpsyg.2021.710493

**Published:** 2021-08-26

**Authors:** Raffael Kalisch, Göran Köber, Harald Binder, Kira F. Ahrens, Ulrike Basten, Andrea Chmitorz, Karmel W. Choi, Christian J. Fiebach, Nele Goldbach, Rebecca J. Neumann, Miriam Kampa, Bianca Kollmann, Klaus Lieb, Michael M. Plichta, Andreas Reif, Anita Schick, Alexandra Sebastian, Henrik Walter, Michèle Wessa, Kenneth S. L. Yuen, Oliver Tüscher, Haakon Engen

**Affiliations:** ^1^Leibniz Institute for Resilience Research, Mainz, Germany; ^2^Neuroimaging Center, Focus Program Translational Neuroscience, Johannes Gutenberg University Medical Center, Mainz, Germany; ^3^Institute of Medical Biometry and Statistics, Faculty of Medicine and Medical Center, University of Freiburg, Freiburg, Germany; ^4^Freiburg Center of Data Analysis and Modelling, University of Freiburg, Freiburg, Germany; ^5^Department of Psychiatry, Psychosomatic Medicine and Psychotherapy, University Hospital Frankfurt, Frankfurt, Germany; ^6^Department of Psychology, Goethe University Frankfurt, Frankfurt, Germany; ^7^Department of Psychology, University of Koblenz-Landau, Landau, Germany; ^8^Department of Psychiatry and Psychotherapy, Johannes Gutenberg University Medical Center, Mainz, Germany; ^9^Faculty of Social Work, Health Care and Nursing Science, Esslingen University of Applied Sciences, Esslingen, Germany; ^10^Department of Psychiatry, Massachusetts General Hospital, Boston, MA, United States; ^11^Psychiatric and Neurodevelopmental Genetics Unit, Center for Genomic Medicine, Massachusetts General Hospital, Boston, MA, United States; ^12^Lee Kum Sheung Center for Health and Happiness, Harvard T.H. Chan School of Public Health, Boston, MA, United States; ^13^Brain Imaging Center, Goethe University Frankfurt, Frankfurt, Germany; ^14^Department of Clinical Psychology and Psychotherapy, University of Siegen, Siegen, Germany; ^15^Department of Psychology, Bender Institute of Neuroimaging, Justus Liebig University, Gießen, Germany; ^16^Department of Public Mental Health, Central Institute of Mental Health, Medical Faculty Mannheim, Heidelberg University, Mannheim, Germany; ^17^Department of Psychiatry and Psychotherapy, Charité Universitätsmedizin Berlin, Corporate Member of Freie Universität Berlin, Humboldt-Universität zu Berlin, and Berlin Institute of Health, Berlin, Germany; ^18^Department of Clinical Psychology and Neuropsychology, Institute of Psychology, Johannes Gutenberg University, Mainz, Germany; ^19^Department of Psychology, University of Oslo, Oslo, Norway

**Keywords:** mental health, adversity, stress, homeostasis, allostasis, adaptation, dynamic system, coping

## Abstract

Resilience has been defined as the maintenance or quick recovery of mental health during and after times of adversity. How to operationalize resilience and to determine the factors and processes that lead to good long-term mental health outcomes in stressor-exposed individuals is a matter of ongoing debate and of critical importance for the advancement of the field. One of the biggest challenges for implementing an outcome-based definition of resilience in longitudinal observational study designs lies in the fact that real-life adversity is usually unpredictable and that its substantial qualitative as well as temporal variability between subjects often precludes defining circumscribed time windows of inter-individually comparable stressor exposure relative to which the maintenance or recovery of mental health can be determined. To address this pertinent issue, we propose to frequently and regularly monitor stressor exposure (E) and mental health problems (P) throughout a study's observation period [Frequent Stressor and Mental Health Monitoring (FRESHMO)-paradigm]. On this basis, a subject's deviation at any single monitoring time point from the study sample's normative E–P relationship (the regression residual) can be used to calculate that subject's current mental health reactivity to stressor exposure (“stressor reactivity,” SR). The SR score takes into account the individual extent of experienced adversity and is comparable between and within subjects. Individual SR time courses across monitoring time points reflect intra-individual temporal variability in SR, where periods of under-reactivity (negative SR score) are associated with accumulation of fewer mental health problems than is normal for the sample. If FRESHMO is accompanied by regular measurement of potential resilience factors, temporal changes in resilience factors can be used to predict SR time courses. An increase in a resilience factor measurement explaining a lagged decrease in SR can then be considered to index a process of adaptation to stressor exposure that promotes a resilient outcome (an allostatic resilience process). This design principle allows resilience research to move beyond merely determining baseline predictors of resilience outcomes, which cannot inform about how individuals successfully adjust and adapt when confronted with adversity. Hence, FRESHMO plus regular resilience factor monitoring incorporates a dynamic-systems perspective into resilience research.

## Introduction

The human brain/mind is a complex dynamic system in constant exchange with its internal (bodily) and external environment. External perturbations of the system in the form of stressors may, if minor, be buffered by homeostatic processes that are located within the system itself or involve recruitment of resources from the environment. There is no lasting change in system function, that is, the coping process leaves the system's mode of operation unaffected. If a perturbation is so strong that it cannot be compensated for by system-environment transactions and exceeds the system's capacity for homeostatic coping, the system will adapt allostatically, by changing its operational set points (Sterling and Eyer, 1988; McEwen and Stellar, 1993; Karatsoreos and McEwen, 2011; Kalisch et al., 2015, 2019). Such allostatic changes can be adaptive, preserving overall system stability—albeit in a new, different mode of functioning—or maladaptive, in which case lasting mental health problems will ensue (McEwen and Stellar, 1993).

Resilience, or the preservation or quick recovery of mental health during and after significant stressor exposure (such as traumatizing events, difficult life circumstances, challenging life transitions, or physical illness; Bonanno et al., 2011; Kalisch et al., 2017), then is the result of successful intra-systemic and inter-systemic adaptation processes (Luthar et al., 2000; Sapienza and Masten, 2011; Rutter, 2012; Kent et al., 2014; Bonanno et al., 2015; Kalisch et al., 2017, 2019). And the key challenge to resilience research is to identify, describe and, ideally, quantify these adaptation processes. Necessarily, this requires longitudinal investigations (Kalisch et al., 2017).

Longitudinal studies aiming to investigate adaptation processes (also “resilience processes” in the remainder) must answer five questions. (i) What is adversity, or stressor exposure, and how is it measured? (ii) How is mental health conceptualized and measured? (iii) On the basis of appropriate stressor and mental health assessments: what, exactly, constitutes a resilient outcome? (iv) What are the potential adaptation processes and how are they measured? and (v) How can the statistical relationship between adaptation processes and resilience as an outcome be established?

In this paper, we will discuss these questions on a fundamental level and, while doing so, try to provide answers that are both generally valid and practical enough to inform concrete resilience study designs. In the process, we build up an example study design and analysis scheme that is modeled on two concrete implementations by the authors (Kampa et al., 2018; Chmitorz et al., 2020a). While this illustrative scenario employs a long observation period of several years as well as regular stressor and mental health monitoring intervals of 3 months, future study designs may well be adapted to accommodate different time frames.

## What is Adversity, or Stressor Exposure, and How is it Measured?

### Definition of Stressor

A stressor is a stimulus or situation that elicits a stress response. The immediate practical problem arising from this definition is that what is a stressor for one person may not be a stressor for another person, or also not for the same person but in a different phase of their life or in different circumstances. Conversely, a stimulus or situation that is entirely harmless for one may be a significant challenge for another. In the face of this, is it possible at all to define an objective stressor or set of stressors to be measured in a resilience study? A further complication is that much of what makes some people resilient to developing mental health problems when confronted with potentially adverse situations presumably lies exactly in the extent and quality of their stress responses to these situations (Kalisch et al., 2015), that is, in whether and in what way the potentially adverse situation really is a stressor to them. Clearly, we cannot afford to neglect this critical type of information. The only way out of this conundrum is that a resilience study must define a set of stimuli/situations that *potentially* are stressors for its subjects and must then assess whether a defined stimulus/situation has occurred or not.

### The Case of Trauma Studies

An example of such an obvious design choice is a frequent type of trauma-resilience studies in which stressor measurement consists in registering a specific pre-defined type of potentially traumatizing life events (LEs) (such as a physical accident or an act of violence) and which then assess the individual differences in mental health responses that occur in the aftermath of the event (Bonanno et al., 2015). Usually, in these studies, the sample only includes subjects in which the event has occurred in the first place. Studies that restrict stressor measurement to one strong potential stressor have the advantage that one can assume that the potential stressor really is a stressor to most study subjects. This means the study will most likely observe a substantial base rate of stressor-induced mental health problems, relative to which resilient responses can be defined, and researchers can be sure to address a significant mental health topic.

The downside of such a design choice is that one cannot exclude that the developing mental health problems that will be observed in some of the traumatized individuals originate from stressors other than the trauma, or that their problems at least only partly relate to the trauma. Resilience, then, in a traumatized subject may well be the consequence of the absence of such additional stressors, which were simply not measured in the study. This would obviously be a trivial explanation that does not necessitate to presuppose any kind of protective, or resilience, processes. An example of a trauma-resilience study where this is a probable explanation is a recent report that individuals' wealth predicts the absence of mental health problems following the onset of physical disability (McGiffin et al., 2019). Wealth prevents or reduces exposure to additional stressors, such as the financial consequences of disability-related medical bills or a diminished household income and, thus, can be used to ease many of the pains and discomforts associated with the disability. Therefore, “resilient” subjects in the study may simply have been less stressor exposed.

### From Studying Trauma to Studying Broader Stressor Exposure

It has been argued earlier that treating a traumatic life event as an isolated stressor is to some extent artificial, as any major stressor is likely to be followed by other stressors (Norris and Elrod, 2006; Kalisch et al., 2015). For example, onset of physical disability may cause financial problems, or the death of a close friend may compromise one's social support network. There is also substantial evidence that LEs can be preceded by stressor exposure, such as when physical or cognitive deficits build up before spousal loss (Vable et al., 2015) or when job insecurity increases before job loss (Kim and von dem Knesebeck, 2016). We therefore proposed that the measurement of stressor exposure in resilience studies should be extended to a wider range of potential stressors and we also argued that these should not only include potential other macrostressors but also seemingly minor events (Chmitorz et al., 2020b) such as daily hassles (DHs) (Hahn and Smith, 1999). The necessity to also consider such microstressors is supported by long-standing evidence that these can also have profound negative mental health consequences, especially when they are many and long-lasting (Serido et al., 2004).

Extending stressor measurement to other macro- as well as to microstressors not only means an extension of the range of stressors to be measured but necessarily also implies a temporal extension. The stressful sequelae of a trauma or also the many different and temporally highly variable stressors associated with more chronic types of adversity or a stressful life transition phase cannot be assessed with a single measurement or over a very short time window only. In fact, the boundaries between traumatic event-like vs. chronic adversity blur under this perspective, as is best illustrated by above example of the potentially traumatizing onset of a physical disability that marks a new life phase with significant chronic burden. Accurate stressor measurement thus requires repeated assessments over a reasonable time interval.

### Example Study

**Figure 1:**
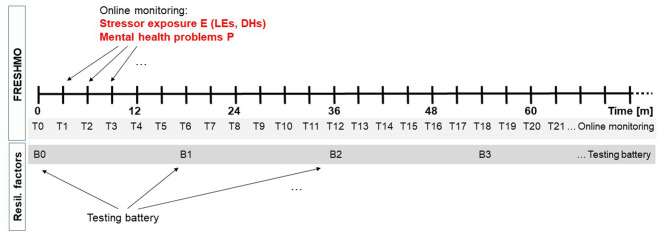
Example design scheme employing the FRESHMO paradigm in combination with repeated assessment of resilience factors. Every 3 months (T1, T2, …), exposure to macrostressors (life events, LEs) and microstressors (daily hassles, DHs) is assessed via self-report using online questionnaires. At the same online monitoring surveys, mental health problems P are reported. Every 1.5 years (B0, B1, B2, …), subjects complete a testing battery for resilience factors.

To illustrate this, we introduce a hypothetical example study where stressor exposure E is measured every 3 months over several years, as seen in Figure 1. For the sake of argumentation, the study may occur in a Western industrialized country in young adults who find themselves in the transition from a familiar environment (family, school) into academic and/or work life, a critical phase of life that we know is associated with a range of new challenges and in many individuals coincides with the onset or exacerbation of stress-related mental health problems. To ascertain a sufficient base rate of mental health problems, subjects may have to meet further inclusion criteria such previous negative LEs, childhood maltreatment, at-risk traits such as neuroticism, or a past mental health diagnosis.

Importantly, the three-monthly stressor monitoring in the study covers both macrostressors (LEs) and microstressors (DHs). Using suitable item lists (Canli et al., 2006; Chmitorz et al., 2020b), we ask subjects to report online on the LEs they experienced in the past 3 months as well as on the DHs they experienced in the past 7 days. For LEs, subjects indicate both the number of occurrences of each event in this interval and the degree to which they perceived the event as straining. For DHs, they indicate the number of days out of the last seven on which a given hassle occurred, as well as how straining it was. Life event and DH item lists are exhaustive (27 and 58 items, respectively), to ideally not miss potential stressors, but they have also been pruned during their development to omit very infrequent and atypical stressors in modern Western societies. In contrast to the retrospective LE reports, subjects' DH reports only cover the past week, because the accuracy of reporting minor stressors will decrease with the length of the reporting time window. We rely exclusively on self-report in our example study because we are not aware of a practicable non-subjective way of measuring a wide range of potential stressors in a time-efficient manner. It is important to note that this makes the study in principle vulnerable to confounding by reporting bias. For instance, subjects may be less inclined to report potential stressors that have objectively occurred to them but did not stress them noticeably; some subjects may have generally more accurate memories or a better verbal understanding of the lists than others; and some subjects may also be inclined not to report stressors that threaten their self-esteem. These biases are limited in our example by choosing relatively short reporting time windows, by giving suitable instructions (emphasizing the necessity for objective and full reporting), and by providing some training in using the instruments. Finally, the distinction that both instruments make between the occurrence of an event or situation (first question for each item) and its perceived severity (second question to be answered only in case the first question was answered in the positive) encourages subjects to report the occurrence also of non-stressful events or situations.

With these design choices, our example study addresses the discussion points made above on how to measure stressors in resilience studies. It is worth emphasizing that the concrete monitoring interval of 3 months is arbitrary and constitutes a trade-off between the needs to maximize sampling frequency and to minimize subject burden and costs. In any concrete study, the trade-off may well be different.

## How is Mental Health Conceptualized and Measured?

Conceptualizations of mental health vary considerably. As classically in psychiatry, they may focus on the absence of symptoms, or mental dysfunctions, or—as in the definition of the World Health Organization (WHO)—on the presence of signs of well-being, competence, and autonomy (World Health Organization, 1951). Another distinction is between a categorical system of classification into defined mental disorders and a transdiagnostic approach that focuses on disturbances in domains of mental and behavioral function and investigates them dimensionally (Craddock and Owen, 2010; Cuthbert and Insel, 2013). Arguably, the choice of concept, construct and tool must depend on the concrete mental health problem a researcher is trying to address in their study. A rationale with generic value, therefore, is to ask which instruments are optimally sensitive for the changes in mental health to be expected in a given sample. In many resilience studies, samples consist in initially healthy subjects of which some develop mental problems over the course of the study. Clearly, dichotomic classification instruments will have limited sensitivity in such samples, and even quantitative instruments used to assess symptoms or dimensions of functioning in a more or less continuous fashion may not necessarily be sensitive for the substantial sub-clinical variation one can expect in typical resilience studies, variation that may nevertheless provide important information on how resilient individuals avoid mental problems (Kalisch et al., 2015). In our example study, we choose a self-report instrument frequently used by general practitioners or other health professionals for the purpose of globally screening for internalizing problems (Goldberg and Hillier, 1979) and which we know is sensitive for sub-clinical and clinical variation and has been extensively employed in work on other primarily healthy populations (e.g., Fleischmann et al., 2020; Jokelaa et al., 2020). We use its total sum score. To match stressor monitoring, subjects also fill in the questionnaire online every 3 months (T0, T1, T2, T3,… in Figure 1), but this interval is also essentially arbitrary and practically motivated.

As in the case of the LE and DH instruments, we emphasize the self-report nature of the instrument. With the advent of objective measures, self-report may well be replaced by more suitable tools.

## What, Concretely, Constitutes a Resilient Outcome?

### The Need to Relate Mental Health Changes to Adversity

The outcome-based definition of resilience as maintenance or quick recovery of mental health during and after adversity (Bonanno et al., 2011; Kalisch et al., 2017; see section Introduction) implies that adversity is necessarily part of the equation. Only registering mental health outcomes without taking into account the adversity a subject was or is exposed to may be informative about mental health, but is not informative about resilience, which in its essence is mental health *despite* adversity (Mancini and Bonanno, 2009). This becomes clear from two hypothetical scenarios in our example study. In scenario 1, two subjects in the study both develop comparable moderate depressive symptoms over the course of their first 2 years of study at university. One of them, subject A, who moved from a small city to a far-away larger city to study and since then struggled with the high costs of living in the region, did not manage to find friends among her peers, was left by her boyfriend (who did not tolerate the long-distance relationship), and failed important exams already in her first term. The other, subject B, was also left by her boyfriend, but already lived in the city before starting her studies and otherwise experienced no major difficulties. At the level of mental health, both subjects react similarly to the initial transition phase into higher education. But given that subject A was exposed to more stressors than subject B, it appears appropriate to classify A as more resilient than B.

In scenario 2, subject A develops much stronger depressive symptoms than subject B, approximately commensurate with her higher stressor exposure. Hence, at the level of mental health, subject A clearly reacts less favorably to her transition than subject B. However, taking into account the differences in stressor exposure now leads to the conclusion that both subjects exhibit similar resilience.

### The Problem of Defining Observation Periods for Unpredictable Stressors

The scenarios raise the immediate question of when during an observation period a resilience outcome is to be registered. It may well be that someone shows initial deterioration of mental health under the pressure of acute challenges, but later, as pressure subsides, recovers. So, both subject A and B in our examples might leave the study after several, say 7, years in perfect mental health and it might then be concluded that they were both resilient to their life transition (although A perhaps more than B because A initially had more stressor exposure than B). However, if one had looked after 2 years, the picture would have been different.

To further complicate matters, it is unlikely that stressor exposure will be restricted to the beginning years of the life transition period that our subjects undergo. One can easily conceive yet another scenario 3, where subject A once more comes under severe pressure toward the end of her university studies, perhaps related to fear of a big final exam, or due to poor professional perspectives in her field of study, or to the onset of a physical disease. This would make her difficult to compare to subject B, who, in this scenario, would again be largely spared from any major adversity. One would also wonder if any deterioration in mental health one might detect in A under the immediate influence of renewed adversity is lasting or only temporary (such that A will recover in the period after the conclusion of the study).

More generally, the challenge in studying resilience to most types of stressors lies not only in the need to measure potential stressors exhaustively and at high frequency and to accurately quantify them (discussed above), but also in the complex temporal patterns in which they can occur. Life is stressful. This complication appears even more in studies where no specific life transition phases are addressed, and it is a general problem faced by any longitudinal study that attempts to realistically assess stressor exposure and associated mental health changes in the way discussed above. The complication is also related to the sheer variety of stressors humans can experience, meaning that one may experience one type of stressor during one period of one's live and another during another period, and this in a multitude of possible combinations of stressors and exposure periods. This makes it difficult (or even impossible) to apply a simple formula for quantifying a person's resilience. For instance, one might be tempted to assess mental health during the beginning and the end of a study observation period (in our example, for instance, years 1 and 7), sum up total stress exposure during the whole observation period, and then simply relate this to changes in mental health (values at year 7 minus values at year 1).

Clearly, this would be problematic. Taking scenario 3, subject A's mental health report at year 7 would presumably be strongly affected by her recently experienced adversities and only to some extent reflect the integral of the stressor exposure that occurred to her over the entire seven years of her study participation. As a result, A would score with strong mental health deterioration from year 1 to year 7. One can easily conceive a third subject C with the quantitatively same stressor exposure as A, but at a different—earlier—time during the study, such that C would already have recovered in year 7. C would then score with less year 1-to-year 7 mental health deterioration than A and be classified as more resilient than A, given that both show the same summed stressor exposure.

**Figure 2:**
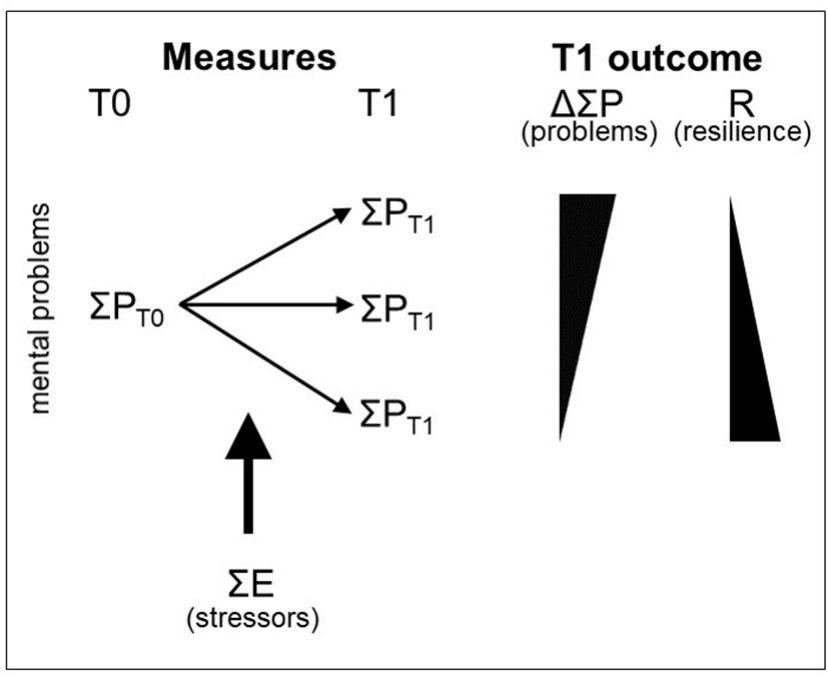
Outcome-based quantification of resilience. Kalisch et al. (2015) proposed to quantify resilience (R) as the ratio of changes in mental health problems P from before (T0) to after (T1) stressor exposure E. Stressors are summed over the observation period (from T0 to T1). Adapted from Kalisch et al. (2015) (their Figure 1). Reproduced with permission.

Given these caveats and pitfalls, how can we apply the formula proposed by Kalisch et al. (2015) for the quantification of resilience (Figure 2)—resilience being understood in conformity with the consensus definition in Kalisch et al. (2017) as a good mental health outcome following stressor exposure—to studies performing longitudinal stressor exposure and mental health monitoring? The formula was explicitly meant by the authors to highlight the general principle of relating mental health changes to stressor exposure, using the simplest possible scenario of two mental health measurement time points bracketing a time window of temporally circumscribed stressor exposure. In the attempt to apply this principle to real-world data, our above considerations call for an approach that takes into account the temporal patterns of stressor exposure and mental health changes in more detail and only then aggregates the information into summary quantities. Our proposal for such an approach is described in the following.

### Residualization-Based Calculation of Stressor Reactivity

**Figure 3:**
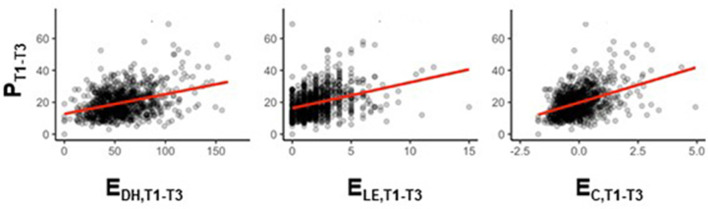
Hypothetical relationship between stressor exposure (E) and mental health problems (P) in the example study. LE and DH counts as well as total mental health problem sum scores P are averaged over the first 3 three-monthly monitoring time points after study inclusion (T0), that is: T1 (month 3), T2 (month 6), and T3 (month 9), to obtain stressor exposure scores E_DH,T1−*T*3_ (left column) and E_LE,T1−*T*3_ (middle column) and mental health problem score P_T1−*T*3_. E_C,T1−*T*3_ is a combined stressor exposure score (mean of E_LE_ and E_DH_ z-scores; right column).

Figure 3 shows hypothetical relationships between the self-reported exposure to LEs (E_LE_) as well as DHs (E_DH_) and subjects' self-reported mental health problems P in the first 9 months of our example study. Life event occurrence is counted by adding up ticked items at any three-monthly monitoring time point before averaging the counts across T1–T3 (E_LE,T1−*T*3_). Daily hassles occurrence is counted is by adding up the numbers of days out of the past 7 that each item occurred, to then also average the counts across T1–T3 (E_DH,T1−*T*3_). Averaging is beneficial in the case of DHs because any monitoring time point only covers DH occurrence in the past week, a time window that might be burdened with too much variability for being a useful representative for the entire preceding 3-months period. (This may not be as relevant for studies with shorter monitoring intervals.) Averaging is in any case advantageous in the case of LEs, given their rare occurrence. Because we average LE and DH counts, we also average P scores, to obtain P_T1−*T*3_. We assume that E and P are moderately positively linearly correlated. (Note that in the absence of a significant relationship between stressors and mental health problems, speaking about resilience would not make sense.) We also have reasons to assume that these E–P relationships can be observed both when only including subjects that have provided all three reports for both variables and when also including subjects that have only provided a minimum of one report in each. The latter allows us to focus analyses on subjects with partial data only, thereby increasing power. Finally, we assume that E_LE_ and E_DH_ scores are well-correlated (expressing the intricate relationship between LEs and DHs discussed initially) and that E_LE_-P and E_DH_-P correlations are in the same range. This permits us to calculate a combined LE/DH stressor exposure score E_C,T1−*T*3_ as the mean of the z-scores of the E_LE,T1−*T*3_ and E_DH,T1−*T*3_. The E_C_-P relationship is more stable in our example study than either E_LE_-P or E_DH_-P relationships, which is why we can focus the further discussion on the combined LE/DH exposure score E_C_.

**Figure 4:**
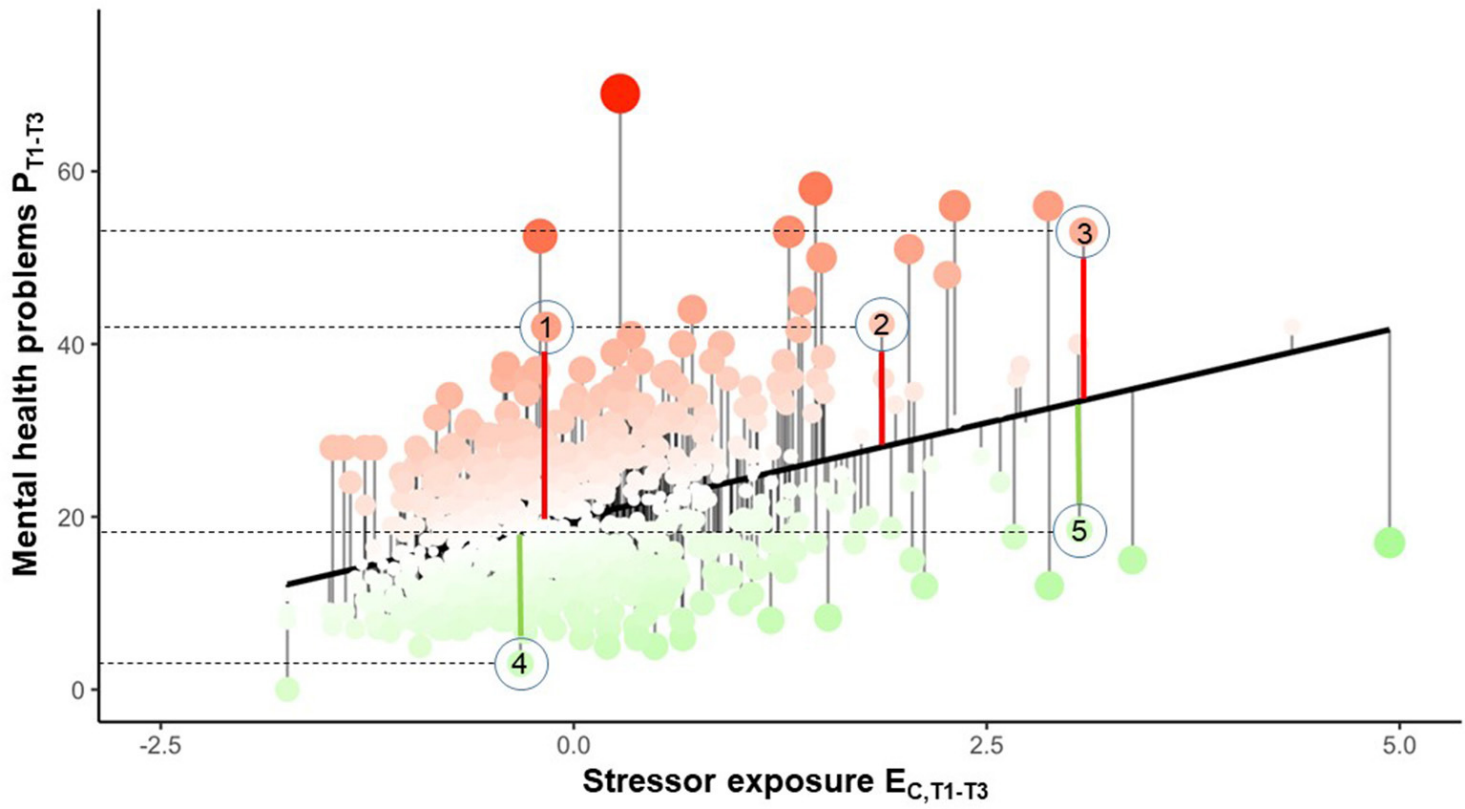
Individual mental health reactivity to stressor exposure (“stressor reactivity”). The regression line shows the normative linear positive relationship between combined exposure to LEs and DHs stressors (E_C,T1−*T*3_) and mental health problems (P_T1−*T*3_) in subjects providing partial data during the first 9 months of the study (monitoring time points T1–T3). The residuals onto the regression line are subjects' deviations from the normative E_C_-P relationship. A strong positive deviation reflects high susceptibility of the subject's mental health to the effects of DHs and LEs (high stressor reactivity, SR) during the chosen time window; a strong negative deviation reflects abnormally low susceptibility (low SR). 1–5 denote arbitrarily chosen subjects.

Given a robust monotone E_C_-P relationship, the distance of an individual's P score to the regression line is likely to be informative about the reactivity of their mental health to stressor exposure in the covered time interval. Figure 4 illustrates the principle of residualizing individual mental health problem scores P on the regression line defined by the group's E_C_-P relationship. The regression line is the normative reactivity of mental health to stressor exposure (in short: “stressor reactivity,” SR) in the whole group during the T1–T3 time window. A subject's residual expresses to what extent the subject deviates from that normal E_C_-P relationship. Individuals with positive residual values (red dots above the line in Figure 4) show comparatively many mental health problems, given their level of stressor exposure; individuals with negative values (green dots below the line in Figure 4) show comparatively few mental health problems, given their stressor exposure (ignoring random variability for the moment). In other words: a positive residual reflects over-reactivity of mental health to stressor exposure (high stressor reactivity, high SR); a negative residual reflects under-reactivity (low SR). In this way, the residual quantifies individual differences in mental health responses to adversity, which to explain is the key objective of resilience research. Because our residual approach employs the group's E_C_-P relationship as the norm, it cannot—and is not intended to—establish a generally valid, sample-independent reactivity measure. Given the idiosyncrasies of any stressor-exposed group and their experienced stressors, the feasibility of a sample-independent resilience measurement has been questioned by us before (Kalisch et al., 2015). Residualization has been introduced to the resilience literature by Amstadter et al. (2014) and van Harmelen et al. (2017).

The residualization approach has the advantage that it inherently corrects for individual differences in the level of stressor exposure. In Figure 4, the same mental health problem score P in two arbitrarily chosen subjects 1 and 2 does not constitute equal degrees of SR, as subject 1 has experienced fewer stressors than subject 2. That is, subject 1 is actually more stressor reactive than subject 2 despite identical P scores. Subject 1's SR, however, is comparable to that of subject 3, who has a higher mental health problem score P but also a higher level of stressor exposure E. Accordingly, subject 1's and 3's residuals (thick red lines) are comparable. Clearly, all subjects lying above the regression line are more stressor reactive than all subjects below the line. Subjects 4 and 5 in the figure have comparable under-normal SR.

### Time Courses of Stressor Reactivity

Low SR during T1–T3 can be interpreted as a first indication of a subject's resilience, in line with our definition of resilience as an outcome, that is, as long-term mental health maintenance despite stressor exposure. A subject with low SR has accumulated relatively less mental health problems across the first nine study months than a subject with high SR, while differences in stressor exposure are controlled for. Of course, 9 months is an arbitrarily chosen time span and hardly reflects a long-term outcome. By increasing the length of the time window, one could make the corresponding SR score more truly reflect a long-term outcome. An individual with consistently low SR over a much longer time span will be mentally healthier despite stressors over that time span than an individual with consistently high SR, provided comparable stressor exposure.

Hence, one possibility would be to build SR scores based on time windows considerably longer than T1–T3. This, however, would neglect a potential—or even likely—dynamic aspect of SR. While it is reasonable to assume that an individual's SR is influenced by trait-like individual characteristics and therefore exhibits some stability over time, it is also safe to assume that one's SR is to some extent affected by temporary internal or environmental factors and therefore not completely constant over time. This is so much more likely as there is increasing evidence that traits, too, can change over time (Soto et al., 2011) and are behaviorally expressed in a contextually dependent and variable way (Matthews, 2018). Thus, SR is likely to show meaningful temporal variation. Another argument that speaks in favor of a more dynamic approach is the temporally variable nature of stressor exposure discussed above. Therefore, for long-term studies, we propose to build individual SR time courses rather than just integrating SR over an entire study time window.

In our example, we do so in sliding windows of temporally overlapping SR scores (T1–T3; T2–T4; T3–T5; …). This approach allows for describing potential temporal fluctuations in SR. At the same time, the use of (moderately) extended and overlapping windows effectively introduces temporal smoothing of the SR time courses and thus reduces spurious fluctuations. The choice in our example study of a sliding time window consisting specifically of three time points is a trade-off between the needs for averaging (see “Residualization-based calculation of SR”) and smoothing on one hand and the wish to retain as much temporal structure in the data as possible on the other hand. This trade-off may look differently in studies with different temporal schedules. Importantly, we use the T1–T3 regression line as the norm for the establishment of residual variations, to make SR scores comparable across time. Hence, the T1–T3 time window effectively provides the norm population for the sample across the entire study period.

**Figure 5:**
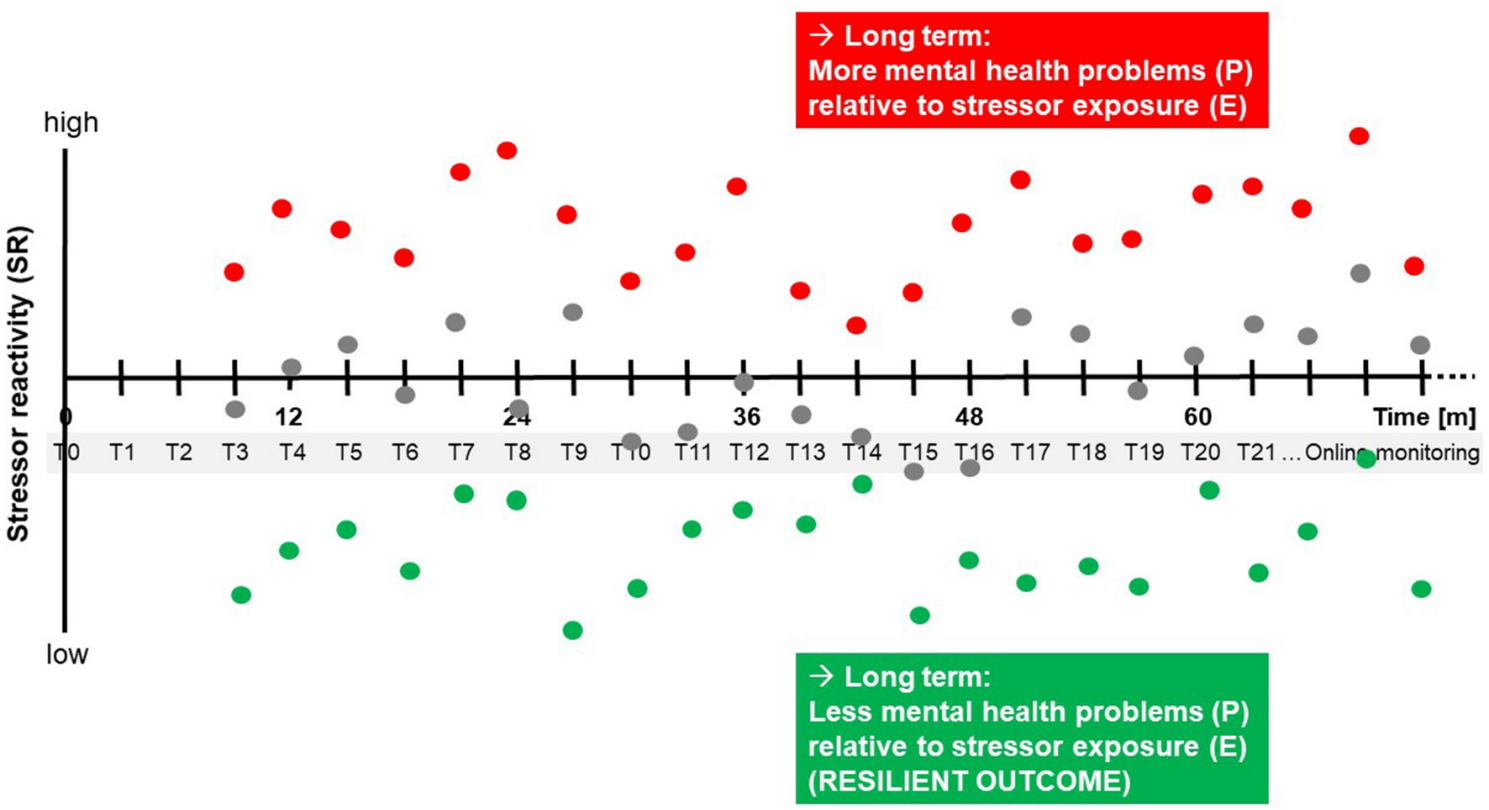
Hypothetical time courses of stressor reactivity. Three hypothetical study subjects with consistently high (red), average (gray), and low (green) stressor reactivity (SR), determined in sliding windows of three consecutive monitoring time points (T1–T3; T2–T4; T3–T5; …), are shown. In the long run, consistently lower-than-normal stressor reactivity leads to fewer mental health problems P relative to individual stressor exposure E, that is, to a more resilient outcome.

The schematic graph in Figure 5 shows SR time courses in three hypothetical study subjects, one with consistently high SR (red), one with consistently normal reactivity (gray), and one with consistently low reactivity (green). After several years of monitoring in the study, the “red” subject will have accumulated more mental health problems relative to his or her stressor exposure than the “gray” and “green” subjects. The gray and green subjects then are more resilient than the red subject, in accordance with the formula for resilience quantification proposed by Kalisch et al. (2015) and shown above in Figure 2 and with the general outcome-based definition of resilience as consensually introduced by Kalisch et al. (2017).

Importantly, however, with SR time courses there is no single time point in the study at which a final resilience outcome is calculated. We thereby respond to the problem highlighted above that stressor exposure in real life is usually not temporally circumscribed and may occur at different times and in different forms in different study subjects. That is, it is impossible in most studies to define a clear time window of stressor exposure relative to which changes in mental health and, thus, a resilience outcome could be determined. This can be considered another advantage that building SR time courses has over a non-dynamic approach.

Based on the SR time course, aggregated quantities might still be calculated, but the original temporal pattern should always be kept as a backdrop. While also alternatives to the proposed sliding-window approach could be devised (e.g., based on differential equations that directly model the impact of stressors on mental health), the time structure should always be taken into account.

## What are the Potential Adaptation Processes and How are they Measured?

**Figure 6:**
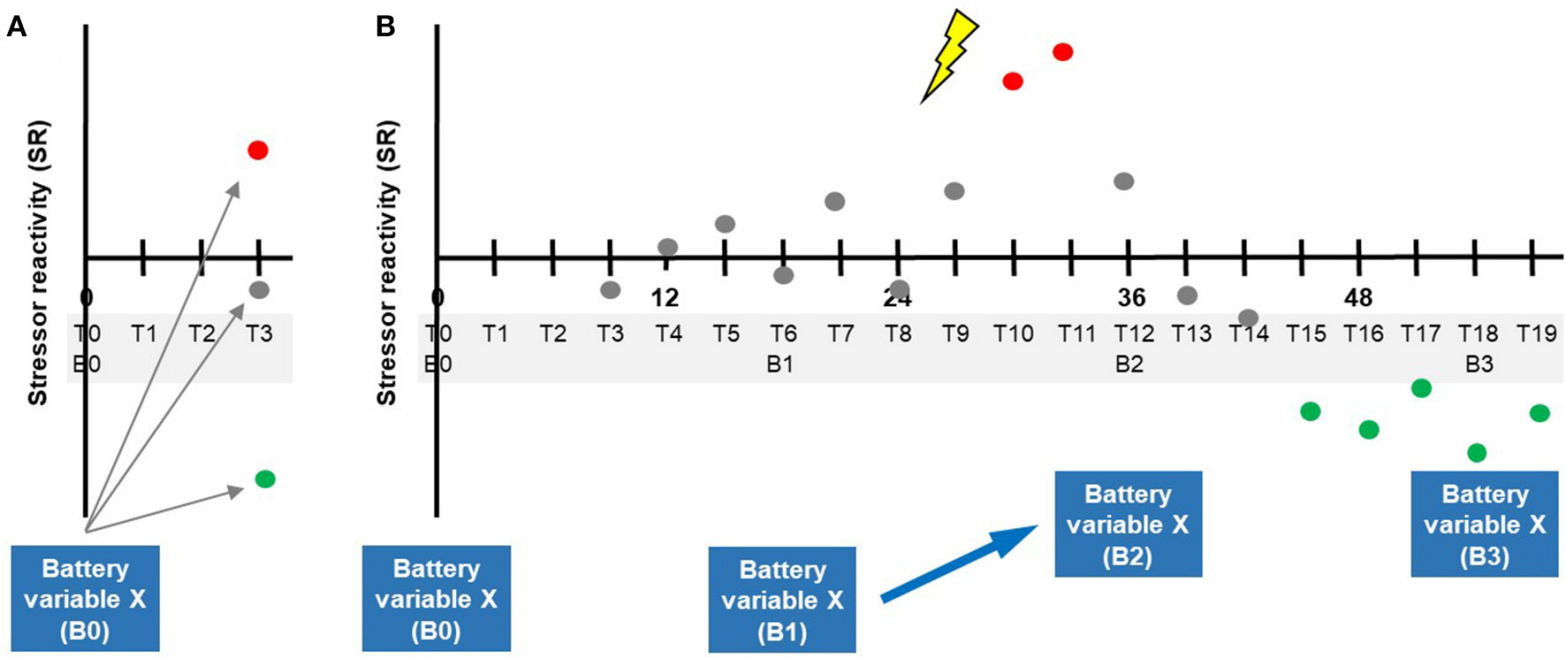
Potential relationships between testing battery measurements and time courses of stressor reactivity. **(A)** Prediction of future SR (here SR_T1−*T*3_) by baseline (B0) battery variable X. **(B)** Hypothetical scenario where, under the influence of significant stressor exposure (lightning bolt), an individual with normal stressor reactivity (gray) improves on a battery measurement X of a resilience factor from B1 to B2 (indicated by the arrow) and where his/her SR decreases (turns green) after an initial increase evoked by the acute stressor effect. This illustrates a lasting change in how the system copes with adversity.

If there is no single way of determining a final resilience outcome in all subjects, this raises the question of what exact dependent variable can be used to identify baseline (T0) predictors of resilience (“resilience factors” that make that outcome more likely) or processes of adaptation (“resilience processes” that effectively generate the outcome). For baseline resilience factors, one might define the first SR score SR_T1−*T*3_ as the outcome variable that serves for calculating prediction analyses, as seen in Figure 6A. This, however, could be considered too short a prediction time window, which is why additional SR scores (T2–T4; T3–T5; …) should be included at a later stage of analysis, e.g., using linear mixed effects models to take into account repeated measurements.

Our emphasis is, however, on the identification of resilience *processes*. We here take advantage of the resilience factor testing battery that is regularly repeated in our example study (see Figure 1). This testing battery is the last crucial element of our generic design proposal. It serves to monitor potential adaptations in any functions or properties (e.g., mental, neural, and bodily) which the authors hypothesize will permit some subjects to stay mentally healthy or to only show temporary mental health impairments despite stressor exposure. Depending on the nature of the hypotheses, it may be a lean tool (e.g., a single online questionnaire assessing a psychological resilience factor) that can be easily added to the regular online stressor and mental health monitoring, or it may consist in more extensive and less frequent tests including those requiring subject presence in a laboratory. In our example study, we choose a 1.5-year testing interval (B0, B1, B2, … in Figure 1), as may be suitable when subject undergo neuroimaging or other biological tests. Life history may be specifically assessed at B0.

Importantly, the battery provides time-dependent predictor variable (X in Figure 6B), i.e., time courses of predictor variables that can potentially explain considerably more variance in SR time courses than a single measurement of any predictor variable at baseline. Time-dependent battery variables that explain significant variance in the SR time courses can then be seen as pointing toward allostatic adaptations in those functions or properties of the brain/mind, the body, or the environment that they index.

### Example of a Resilience Process

To illustrate the distinction between resilience factors and resilience processes and their relation to SR time courses, we build on a simple scenario employed by Kalisch et al. (2019) for that purpose. We assume that volitionally regulating emotions away from negative toward more positive emotional states using verbal strategies of reappraising the meaning of, or reframing, potentially threatening situations (“positive reappraisal”; Lazarus and Folkman, 1984; Gross, 1998) protects mental health in stressor-exposed individuals. Cognitive positive reappraisal exerts this assumed effect by dampening stress reactions and thereby limiting the expense of resources (time, energy, cognitive capacity, financial, or social capital) that accompanies them and that can be deleterious to body and mind if the stress reactions are too intense and too frequent. Hence, every time an individual is confronted with a significant stressor that more or less automatically induces a negative emotional state and then reappraises the situation in a benign way (while also avoiding unrealistically positive appraisals), he or she will save resources and make mental health impairments less likely to occur (Kalisch et al., 2015). (In the terminology introduced in Kalisch et al., 2015, the concrete act of performing reappraisal is a resilience mechanism.)

Individuals with a good ability and habitual tendency to use reappraisal for emotion regulation are more likely to regulate their emotions using reappraisal (to employ the resilience mechanism that they have at their disposal), and it can be hypothesized that any good measure of reappraisal ability and tendency taken at T0 will therefore negatively predict SR_T1−*T*3_ and later SR time windows. (Such measure may be a questionnaire or a laboratory reappraisal task or perhaps an index of neural activity associated with a reappraisal task.) That is, better reappraisers will be less stressor reactive during the first nine or more study months. If this is correct, individual reappraisal ability/tendency can be considered a resilience factor.

If the measurement of reappraisal ability/tendency (resilience factor) is repeated regularly (i.e., with every application of the testing battery B0, B1, B2, …), this can also inform about reappraisal as a process. Two resulting patterns are conceivable. An individual may not change his or her reappraisal ability/tendency much over time, that is, may just stay a more or less good or bad and frequent or infrequent reappraiser. Good reappraisers should show less SR than bad reappraisers also at monitoring time points past T3, and the inter-individual variance of SR time courses explained by the repeatedly measured reappraisal ability/tendency should increase, relative to a single measurement of reappraisal ability/tendency only at T0, simply because repeated measurements provide more reliable information on individual reappraisal ability/tendency. From such an apparent dampening effect of reappraisal ability/tendency (factor) on SR in good reappraisers one can then infer, indirectly, that these individuals presumably successfully use reappraisal in their daily lives (process) and that this suffices them to maintain emotional balance and good mental health. That is, the external perturbations that the system experiences are not strong enough to exceed the system's capacity for coping, and, consequentially, the system does not lastingly change its way of functioning. The individual copes as it is used to cope. In the terminology introduced at the start of this paper, coping in this case is homeostatic, and real-life use of reappraisal is a homeostatic resilience process.

The situation is different when an individual's reappraisal capacity is not sufficient to maintain stability. In this case, the system will have to adapt in an allostatic way, i.e., it will struggle to find new or better ways of coping that constitute lasting shifts in system function. For example, important life challenges may be answered by greater reappraisal efforts and, if successful, this may make an individual a better reappraiser than he or she was while life was still less challenging (see the scenario in Kalisch et al., 2019, their Figure 7). In the testing battery, such kind of training effect should become apparent in higher values on the reappraisal ability/tendency predictor variable X, along with the variable well-explaining variance in SR. In Figure 6B, a hypothetical time course is shown where, under the influence of significant stressor exposure (lightning bolt), an individual with normal SR (gray) improves on reappraisal ability/tendency (battery variable X) and where his or her SR decreases (turns green) after an initial increase evoked by the acute stressor effect. Thus, the example illustrates a lasting change in a measure of a resilience factor that indicates an allostatic process of adaptation. Hence, as opposed to above example of real-life reappraisal use as a homeostatic resilience process, allostatic resilience processes occur at a higher level of system function, by constituting shifts in the way the system operates.

It is, of course, also conceivable that an important life challenge will not be answered by the organism with an increase specifically in reappraisal ability/tendency, but maybe in some other coping function or mechanism. This would also be an allostatic resilience process and should become apparent in the testing battery, provided the existence of battery variables that index that function.

Finally, the system may also not succeed in adapting well to the situation. This should become apparent in lasting increases in SR as well as potentially in decreases in testing battery variables that index resilience factors. (One might give up using reappraisal and become an even worse reappraiser than before; in turn, variables associated with pathological forms of coping, e.g., catastrophizing or rumination, might increase.) The latter scenario would also constitute an allostatic process, but a maladaptive one, which should accordingly be classified as pathological or pathogenic, as opposed to the adaptive resilience processes discussed before.

Hence, a combination of repeated measurements of SR with repeated battery measurements of system functions (including hypothesized resilience factors) can help identify both homeostatic and allostatic resilience processes.

## How Can the Statistical Relationship Between Adaptation Processes and Resilience as an Outcome be Established?

Next to the use of reliable measures, the successful identification of resilience processes will depend on the availability of suitable mathematical methods to link resilience predictors and outcomes. There are multiple ways to assess the statistical relationship between changing individual properties, as measured in battery variables X, and resilience, as assessed using changing SR scores. A rather simple approach would draw on the literature on dynamic predictions in clinical settings (Putter, 2013) and apply it to the smoothed SR scores. Such an approach might predict the following SR scores either with the latest baseline information or a function of all previous baseline information, which allows for taking the history of adaptation processes into account. More specifically, each smoothed mean SR score (T1–T3; T2–T4; T3–T5; …) is predicted with (the latest) baseline information of B0, B1, or later. The temporal sequence of regression coefficients β1, *T*1 − *T*3 − β1, *T*3 − *T*5 of the same predictor X can be connected and, for instance, be smoothed to reflect the idea of slow changes and potentially increasing analytical power. Statistical significance can be assessed with appropriate standard errors taking the repeated measures into account. Algorithms for variable selection can assist the search for resilience factors in big baseline batteries (Schmidtmann et al., 2014; Zöller et al., 2016).

In case the resilience factor changes over time, its prediction capabilities might be reduced the more time lies between the monitoring time point T and the latest administration of the testing battery B. This “aging” of predictors can be attenuated by modeling the trajectories of the resilience factors, potentially taking the stressor load and mental health into account.

Regression-based approaches are limited in that they assume uni-directional causality (resilience factors influence the SR score but not vice versa). However, there are other models which treat each single observation of P, E, and the battery assessments of potential resilience factors X as samples from continuous trajectories. Such dynamic models, which can, for instance, be based on differential equations, take into account the (inferred) value of P, E, and the resilience factors at every point in time. They require and benefit from domain expertise, since every trajectory affects the change of itself and all other variables according to a predefined system of equations. Their estimated coefficients can be also tested for significance (Raue et al., 2009). Such models allow for irregularly sampled measures and, accordingly, are able to bridge disparate temporal resolutions as well as entirely missing observations (Köber et al., 2020).

## Discussion

### Limitations and Comparison With Other Approaches

While our generic study design, involving frequent and extensive stressor monitoring, reduces the likelihood that we miss stressors with significant mental health impact, we can of course not exclude this. Our monitoring method is an approximation to the aim of complete stressor measurement.

A potential criticism of the proposed SR scores might be that they are also calculated for individuals with very low stressor exposure E (left end of the regression line in Figure 4), and that resilience in the absence of significant adversity is not a meaningful concept (Mancini and Bonanno, 2009). This criticism, however, would fail to consider that stressor exposure may well change over the course of a study and that a subject with initially low stressor exposure might well experience more stressors later. This means that excluding a subject from the calculation of SR scores based on a low E score at a given time point would unnecessarily exclude that subject from the long-term observation needed to determine resilience. Further, given the assumed monotone E–P relationships (Figure 3), any such decision to exclude a subject would have to be based on an arbitrary E threshold that is not anchored to any kind of turning point or “true” threshold present in the stressor exposure data.

One could still argue that subjects showing consistently low E scores over the course of a study may better be excluded, to thus fulfill the reasonable criterion that adversity has to be present. We therefore propose as a general rule for the analyses of SR data that (a) primary analyses based on the entire samples be complemented by secondary analyses of those two-thirds of the subjects with the highest overall stressor exposure E, and that (b) the results of those secondary analyses should go in the same direction as those of the primary analyses, in order for the primary analysis results to be considered valid.

Another criticism may be the combination of micro- and macrostressors in a common E_C_ score. Although the singular impact of any given microstressor on mental health is presumably clearly different from (smaller than) the impact of any macrostressor, micro- and macrostressors are tightly related, as described above. Yet there is, to this date, no universal quantitative way to reliably describe their relation, let alone to do so in a longitudinal fashion. Hence, using the mean of the z-scores of the DH and LE counts may as of now be the best practical solution. This will have to find empirical support from future studies allowing us to test if E_C_-P relationships are indeed consistently more stable than either E_LE_-P or E_DH_-P relationships.

We emphasize again that the use of the SR score-based method presented in this paper is not limited to the concrete choice of stressor and mental health instruments made in the example study. The only restriction inherent in the method is that the chosen E score must explain sufficient variance in the chosen P score such as to avoid quasi-identity of the resulting SR scores with subjects' P scores. [In the extreme case of a zero E–P correlation and identical SR and P scores, predictors of resilience (SR) and mental health (P) would be identical, too, and it would be meaningless to apply the concept of resilience—mental health *despite* adversity—to the data.] It is also worth pointing out that one can theoretically also build several different types of SR scores from the same data set, such as when using multiple or multi-factorial mental health instruments, each of which can be separately related to stressor exposure, or when using item-level partial least squares regression approaches that allow for mapping the space of stressor and mental health items onto a smaller number of latent components, each of which expresses the reactivity of specific mental health domains to specific stressor domains (e.g., Schüler et al., 2020). Such approaches can be used to differentiate resilience factors or processes associated with specific types of outcome-based resilience, as defined by different combinations of types of adversity and mental health consequences. One might thereby be able to identify dysfunction-specific as well as dysfunction-independent (general) resilience factors/processes or even show that some resilience factors/processes protect against any type of mental health problem caused by any type of adversity (global resilience factors/processes) (Kalisch et al., 2015; see also Ayash et al., 2020).

To exclude potential misunderstandings, we would like to emphasize that SR-based trajectories differ fundamentally from the type of mental health trajectories that have been prominently used in resilience research to delineate mental health responses to potentially traumatizing events (e.g., loss of a spouse, stroke, …) or to onset of chronic adversity (e.g., physical disability, chronic pain, …) (see above and Bonanno et al., 2011). Although in some cases these studies have succeeded in controlling for the level of stressor exposure at the start of the trajectory (by assuring that the severity of the traumatic event or of the chronic adversity whose onset defines trajectory start is comparable across the cohort), they nevertheless do not take into account stressors occurring after trajectory start. As discussed above, such stressors may include the potentially individually very different sequelae of a trauma or some chronic type of adversity resulting from the trauma. These studies can therefore not exclude that shifts in mental health often observed in these cohorts, whether from good to bad or vice versa, are caused merely by individual differences in stressor exposure. By contrast, SR trajectories as introduced here inherently take such potential influences into account.

In a recent paper, we have proposed a general framework for an approach to determine resilience processes based on a dynamic network account of psychiatric disorders and resilience (Kalisch et al., 2019). Both that and the current account converge in that they rely on frequent repeated measurements of stressor exposure, mental health problems, and potential resilience factors and in that they consider these to be usually time-variant. The network approach differs from the current approach in that it builds on the quantification of temporal interactions between and within stressor-induced mental health problems and asks how these are modulated by selected resilience factors. The current approach, by contrast, ignores interactions of mental problem and combines problems and stressors into a single quantity (SR), which it attempts to predict by resilience factors. It thus sacrifices a fine-grained analysis of mental health problems and their dynamics for the possibility to potentially assess the predictive influence of a larger number of resilience factors and may therefore also be more suitable for multi-factorial and exploratory approaches. The two approaches can thus be seen as complementary. Also, other approaches for dynamic modeling, such as differential equations, might be considered. These alternative routes, however, are still considerably more conceptual than the current account at this stage of methodological development and await concrete mathematical formulation. By contrast, our proposed sliding-window residual approach affords a way to already now test resilience factors and resilience processes in existing data.

## Outlook

As resilience research is moving from studying resilience-conducive traits to studying malleable and time-dependent resilience factors and from identifying baseline resilience predictors to characterizing processes of adaptation, the development of suitable study designs, analytical concepts and associated mathematical methods becomes a crucial field of methodological research. The present paper aims to enrich the current debate and to propose a concrete solution, which we believe proposes important novel elements: a study scheme involving the high-frequent concurrent measurement of micro- and macrostressors in combination with repeated measurements of mental health [Frequent Stressor and Mental Health Monitoring (FRESHMO)-paradigm]; the accompanying repeated measurement of potential resilience factors in a testing battery; a way to quantify resilience as a dynamically changing outcome; and a way to link resilience factors with the outcome in a dynamic fashion, to thus identify resilience processes. We consider our proposal a generic solution that can serve as a blueprint for future resilience studies, notwithstanding necessary adaptations to the concrete context (e.g., by choosing other measurement and sliding-window intervals, by using stressor assessment instruments that are better suited for the population of interest, or by measuring mental health with different tools). We expect that our approach will be particularly fruitful in populations and life situations where individual change, that is, allostatic adaptation, is an important element of mental health preservation. This can be almost certainly assumed for the life transition phase into adulthood that we chose here as object of our example study, but we assume that any kind of confrontation with significant change and adversity will be accompanied by similar adaptation phenomena. We are thus confident that this and other dynamic approaches will considerably advance the field of resilience research.

Future work may also address whether our approach can be extended to other outcomes than mental health. For instance, outcomes such as academic success or social competence are important criteria in developmental resilience research while aspects of functioning are frequently in the focus of research on diseased or old-age populations.

In situations where longitudinal repeated assessment is not possible for practical reasons, an alternative could be to retrospectively assess *changes* in mental health that a subject may have experienced over some (limited) past time window and to also assess stressor exposure within the same retrospective time window. Single quantity scores calculated based on such data can then at least inform about mental health reactivity to stressors within the chosen time window and may be tested for their association with concurrently measured resilience factors. An example is a recent study on factors negatively associated with mental health deteriorations induced by the COVID-19 pandemic as assessed during the first Corona lockdown for a short past time window of several weeks (Veer et al., 2021). In studies where the relevant stressor exposure lies in the more remote past—such as in investigations on the long-term effects of early-life stress—suitable study designs may also involve relating current mental health problems, or deteriorations in mental health, to this past stressor (e.g., van Harmelen et al., 2017). Such residual scores could then be interpreted as expressing later (adolescent or adult) mental health reactivity to childhood adversity and in turn be linked to current or past protective factors.

## Author's Note

A previous version of this manuscript has been published as a preprint (psyarxiv.com/jg238/).

## Author Contributions

All authors contributed to the conceptualization and discussion of the principles of operationalizing resilience and designing longitudinal resilience studies and to the editing of the manuscript. RK, OT, and HE wrote the manuscript.

## Conflict of Interest

RK receives advisory honoraria from JoyVentures, Herzlia, Israel. The authors declare that the research was conducted in the absence of any commercial or financial relationships that could be construed as a potential conflict of interest.

## Publisher's Note

All claims expressed in this article are solely those of the authors and do not necessarily represent those of their affiliated organizations, or those of the publisher, the editors and the reviewers. Any product that may be evaluated in this article, or claim that may be made by its manufacturer, is not guaranteed or endorsed by the publisher.
